# PEEK Interference Screws Show Significant Tunnel Enlargement After ACL Reconstruction and is Comparable to Adjustable-Length Loop Cortical Button Fixation

**DOI:** 10.1007/s43465-023-01029-8

**Published:** 2023-11-19

**Authors:** Christian Coppola, Sandra Krost, Armin Runer, Christoph Raas, Bernhard Glodny, Raul Mayr

**Affiliations:** 1 Department of Orthopaedics and Trauma Surgery, Medical University Innsbruck, Innsbruck, Austria; 2 Department of Radiology, Medical University Innsbruck, Innsbruck, Austria

**Keywords:** ACL reconstruction, Anterior cruciate ligament, PEEK interference screws, Cortical button fixation, Tunnel enlargement

## Abstract

**Background:**

It is unclear whether the use of polyetheretherketone (PEEK) interference screws for anterior cruciate ligament (ACL) reconstruction leads to postoperative tunnel enlargement. Femoral tunnel enlargement was further compared with adjustable-length loop cortical fixation.

**Methods:**

Eighteen patients with ACL reconstruction using hamstring grafts were retrospectively divided into two groups. Eleven patients were treated with the ACL reconstruction technique using a PEEK interference screw for femoral graft fixation. Seven patients received adjustable-length loop cortical buttons for femoral fixation. Tibial ACL graft fixation was performed using PEEK interference screws. Tunnel volume changes were assessed using computed tomography (CT) scans performed after surgery (100%) and after 1 year. The maximal tunnel diameter was measured.

**Results:**

The group with femoral screw fixation showed a mean tunnel volume change of 108.15 ± 13.7% on the tibial side and 124.07 ± 25.38% on the femoral side. The group with femoral button fixation showed a tunnel volume change of 111.12 ± 12.72% on the tibial side and 130.96 ± 21.71% on the femoral side. The differences in femoral tunnel volume changes were not significant (*P* = 0.562). Femoral tunnels with PEEK screw fixation showed significantly larger diameter after 12 months in comparison with button fixation (13.02 ± 1.43 mm vs. 10.46 ± 1.29 mm, *P* < 0.001).

**Conclusions:**

PEEK interference screws were associated with significant tibial and femoral tunnel enlargement. Femoral tunnel enlargement was comparable between PEEK interference screws and button fixation. Final femoral tunnel diameter was significantly larger with PEEK screw fixation in comparison to button fixation.

## Introduction

Tunnel enlargement following anterior cruciate ligament (ACL) reconstruction is a well-known and often-observed phenomenon [1–3]. It has been shown that radiographic evidence of tunnel enlargement does not directly correlate with clinical outcomes [4]. However, enlarged tunnels may have significant implications for graft fixation and graft incorporation in revision ACL reconstruction [5]. In cases of severe tunnel enlargement, two-stage revision surgery with primary bone tunnel filling may be necessary [1].

The etiology of tunnel enlargement following ACL reconstruction is multifactorial [3]. In brief, both mechanical and biological factors cause changes in the postoperative tunnel volume [1, 2, 4]. Biological factors involved include tissue reactions at the healing surface between graft and bone. Mechanical factors mainly include micromovements at the tendon–bone interface. Several studies have investigated the relationship between tunnel enlargement and fixation techniques in ACL reconstruction [6, 7]. With suspensory graft fixation using cortical button devices, no foreign material is left in the bony tunnel, which may be beneficial for graft incorporation [8]. On the other hand, cortical button fixation has been shown to allow graft micromotion [2, 9, 10]. Adjustable-length loop cortical button devices were designed to avoid intraoperative button loop measurement and calculation, but biomechanical studies suggest they are associated with greater loop elongation in comparison with fixed-loop button devices [9, 11]. Tunnel enlargement after cortical button fixation for ACL reconstruction is been reported [2].

Aperture graft fixation close to the joint [12] may reduce graft micromotion within the tunnel [10], but the foreign material in the tunnel reduces the tendon-to-bone ingrowth area. It was at one time thought that biodegradable material would be ideal for interference screws in ACL reconstruction, but it was later confirmed that although biodegradable screws were associated with good clinical outcomes [13], full resorption was only seen during the long-term follow-up [1, 14]. In addition, problems with severe tunnel enlargement due to osteolysis, foreign-body reactions, and intraoperative screw breakage have been reported in the literature [14, 15]. Interference screws made of polyetheretherketone (PEEK) have, therefore, become increasingly widely used in recent years. PEEK is an exceptionally strong thermoplastic polymer [16] that offers a high degree of biocompatibility and biostability. In contrast to metal interference screws, PEEK screws do not produce any artefacts on computed tomography or magnetic resonance imaging scans [16, 17]. It is claimed that due to its mechanical and nonmechanical properties, PEEK is suitable for both short-term and long-term implantation in human tissue. Results for osteointegration and biocompatibility of PEEK materials in human bone have mainly been reported in spinal and dental surgery [18]. There have only been a few studies investigating the behavior of PEEK interference screws in ACL reconstruction [17, 19]. In a previous study by Shumborski et al. [19], tunnel volumes measured on magnetic resonance imaging (MRI) of PEEK interference screws and titanium screws appeared to be equivalent 12 months after the operation. Lind et al. [17] reported that no tunnel widening was observed when open-architecture PEEK interference screws were used in ACL reconstruction, but the diameter data were not explicitly reported. In contrast, Uzumcugil et al. [20] reported significant tibial tunnel enlargement in measurements on plain radiographs when PEEK interference screws were used for tibial ACL graft fixation.

Volumetric tunnel investigations on computed tomography (CT) scans may represent the most accurate measurement method for measuring tunnel enlargement [21]. CT scanning is the gold standard and provides greater precision in comparison with measurements based on MRI or plain radiography [1, 22]. There have been no studies reporting the use of volumetric CT measurements of tunnel enlargement after ACL reconstruction with PEEK interference screws. Nor has femoral tunnel enlargement after ACL reconstruction with PEEK interference screws and adjustable cortical button fixation so far been compared.

The aim of the present study was to investigate changes in tibial and femoral tunnel volumes after ACL reconstruction using PEEK interference screws. Changes in the femoral tunnel volume were also compared with adjustable-length loop cortical fixation. It was initially hypothesized that PEEK interference screws would not be associated with any tunnel enlargement 1 year postoperatively. The second hypothesis was that femoral PEEK interference screw fixation would lead to less tunnel enlargement in comparison with adjustable-length loop cortical button fixation.

## Methods

### Patients

The study was approved by the local ethics committee. An informed consent has been obtained from all patients and involved persons.

CT data from 18 patients were analyzed retrospectively from a prospective study cohort. As we routinely don`t perform postoperative CT scans after ACLR, we used data from another prospective study with different measurement outcomes to measure tunnel enlargement. Patients aged 18–41 years undergoing ACL reconstruction using autologous hamstring grafts were included. CT scans of the knee were performed within 3 days after surgery and 1 year postoperatively. Fermoral ACL graft fixation was chosen on the basis of the surgeons’ preferences. In relation to ACL graft fixation techniques, two groups were defined retrospectively. Group A consisted of 11 patients in whom the ACL reconstruction technique was performed using a PEEK interference screw (PEEK Interference Screw; Arthrex, Inc., Naples, Florida, USA) for tibial and for femoral graft fixation. Group B consisted of seven patients in whom ACL graft fixation was performed using a PEEK interference screw for tibial graft fixation and an adjustable-length loop cortical button (TightRope RT; Arthrex, Inc., Naples, Florida, USA) for femoral fixation. Inclusion criteria were complete ACL rupture on magnetic resonance imaging and clinical instability. Exclusion criteria were total collateral ligament rupture or significant meniscus injury requiring extensive resection (> 50% Meniscus tissue resection) or repair leading to a change of the postoperative rehabilitation protocol.

### ACL Reconstruction — Surgical Technique

ACL reconstructions were performed by different surgeons trained in ACL reconstruction.

### Graft Preparation

After harvesting, the semitendinosus and gracilis tendons were prepared on a preparation table. The free ends of the graft were whipstitched using a nonresorbable suture (FiberWire; Arthrex, Inc.). The tendons were folded to obtain a four-stranded tendon graft. For button fixation, the tendons were folded over an adjustable-length loop cortical button device (TightRope RT; Arthrex, Inc., Naples, Florida, USA).

### Screw Fixation

Femoral tunnel was drilled through the anteromedial portal at the center of the femoral ACL insertion site at 120° knee flexion. The femoral tunnel was 25 mm long. The mean drilled femoral tunnel diameter was 8.2 ± 0.5 mm. The graft was fixed using a 23-mm long PEEK interference screw with a diameter 1 mm less than the tunnel diameter.

For the tibial insertion, a full tunnel was created using a drill guide placed at the tibial ACL stump. A full tibial tunnel was drilled, preserving the native tibial ACL stump. The mean drilled tibial tunnel diameter was 8.8 ± 0.6 mm. The knee was cycled approximately 10 times for graft preconditioning. The graft was fixed at 30° of flexion by inserting the PEEK interference screw into the tibial tunnel aperture. The screw diameter selected was 1 mm larger than the tunnel diameter, and the screw length was 28 mm*.* An additional extracortical fixation was performed in four of 11 patients by knotting the graft sutures over a suture device (Suture Washer, Smith & Nephew.).

### Button Fixation

The femoral tunnel was prepared for adjustable-length loop cortical button fixation. A 4.0-mm tunnel was drilled through the anteromedial portal at the center of the femoral ACL insertion at 120° knee flexion. This tunnel was overdrilled to obtain a socket tunnel of 25 mm. The mean drilled femoral tunnel diameter was 7.4 ± 0.7 mm. After femoral graft fixation, the knee was cycled approximately 10 times. Tibial tunnel creation and graft fixation were identical to the technique described above for the screw group. After tibial graft fixation, the graft was additionally tensioned by shortening the adjustable loop of the femoral button at 30° of flexion. An additional extracortical tibial fixation was performed in six of seven patients by knotting the graft sutures over a suture device (Suture Washer, Smith & Nephew).

### Rehabilitation

From the first day, patients engaged in active quadriceps exercises and passive knee motion, supported and guided by trained physiotherapists. The patients were allowed walking with full weight-bearing immediately. A knee brace was worn for two weeks postoperatively. Cycling, swimming, and muscle training were permitted from fourth week. Running was allowed after 12 weeks. Other full-activity sports, as well as pivoting sports, were allowed after 6–9 months from the operation.

### Radiological Measurement

Multidetector CT scanning (GE Discovery CT 750 HD; GE Healthcare, Chicago, Illinois, USA) was performed using a slice thickness of 0.625 mm (512 × 512 voxels). Images were acquired at 100 kV and 120–400 mAs, with a noise index of 25. Multidetector CT images were used for tunnel diameter and volume measurements. Tunnel diameters in millimeters and volumes in cubic centimeters within the first week postoperatively (Defined as 100%) and 1 year postoperatively were compared between the two study groups. Bone tunnel volume was measured on the axial slices. In the group with PEEK interference screw fixation, the screw volume was included in the measurement. The cross-sectional area of the bone tunnel was added up and multiplied to calculate the total volume on every third slice (AW Server 2.0; GE Healthcare). The interrater intraclass correlation coefficient (ICC) with this measurement technique has been reported to be between 0.606 and 0.922 [2].

For diameter measurements, the images were orientated along the longitudinal axis of the tunnel, and the maximum diameter was measured. All of the measurements were performed by the same investigator (board certified radiologist, B.G.).

### Statistical Analysis

Statistical analysis was performed using IBM SPSS Statistics for Windows, version 27.0 (IBM Corporation, Armonk, New York, USA).

The chi-squared test was used to investigate differences in categorical data. Parametric data are presented as means with standard deviation (SD). Data were tested for normal distribution using the Kolmogorov–Smirnov test. Student’s *t* test was used to compare normally distributed data. Changes in tunnel diameter and volume are presented in percentages, as means with standard deviation. Reported *P* values are two-sided, with significance set at < 0.05.

## Results

### Baseline Data

Relative to the baseline data, there were no significant differences between the two groups with regard to mean age at the time of operation (*P* = 0.746) or sex (*P* = 0.629) (Table 1). In the button fixation group, the drilled diameter on the femoral side was significantly smaller than in the screw fixation group (*P* = 0.035). Two partial meniscus resections in the white–white zone were performed in the PEEK group; one disc meniscus was trimmed to physiological shape in the button fixation group (Table 1).

**Table 1:** Baseline data (mean with standard deviation) for a prospective group of patients with ACL reconstruction with PEEK interference screw fixation or cortical button fixation

	Screw fixation (*n* = 11)	Button fixation (*n* = 7)	*P* value
Age (y)	27.18 ± 6.65	28.29 ± 7.39	0.746
Sex			
Female	5 (45%)	4 (57%)	0.629
Male	6 (55%)	3 (43%)	
Drilled diameter			
Femoral	8.18 ± 0.56	7.42 ± 0.8	0.035
Tibial	8.8 ± 0.63	8.42 ± 0.58	0.248
Partial meniscus resection	2	1	

### Tunnel Volume and Volume Change

Twelve months after surgery, the group with PEEK interference screw fixation showed tunnel volume enlargement of 108.15 ± 13.7% on the tibial side and 124.07 ± 25.38% on the femoral side.

**Fig. 1:**
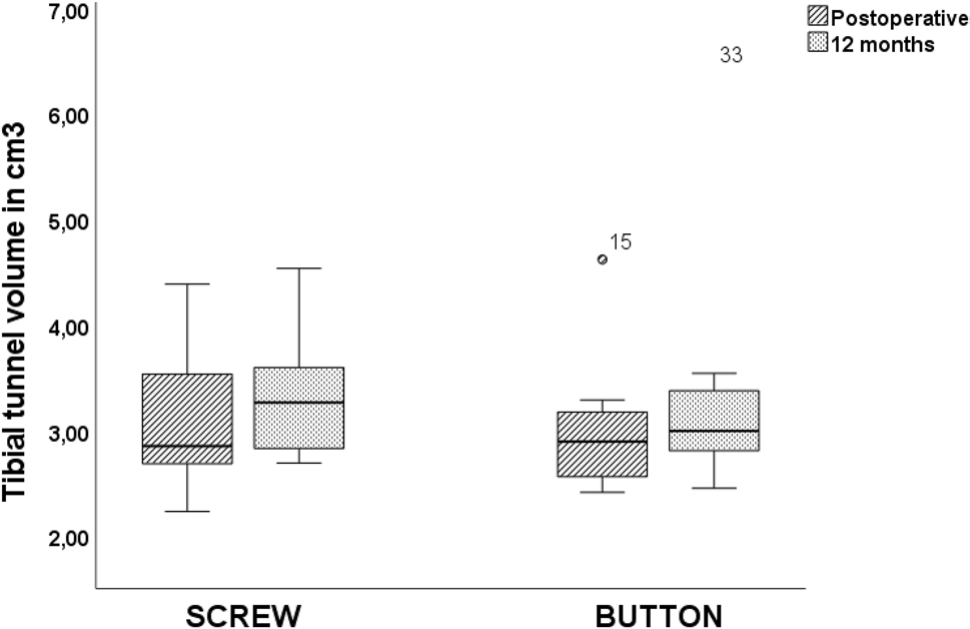
Box plots with medians and interquartile ranges (25–75%) showing tibial tunnel volume in cubic centimeters in the two groups (PEEK, button) in CT scans at different time points (postoperative, 12 months). It should be noted that PEEK interference screws were used for tibial fixation in both groups

**Fig. 2:**
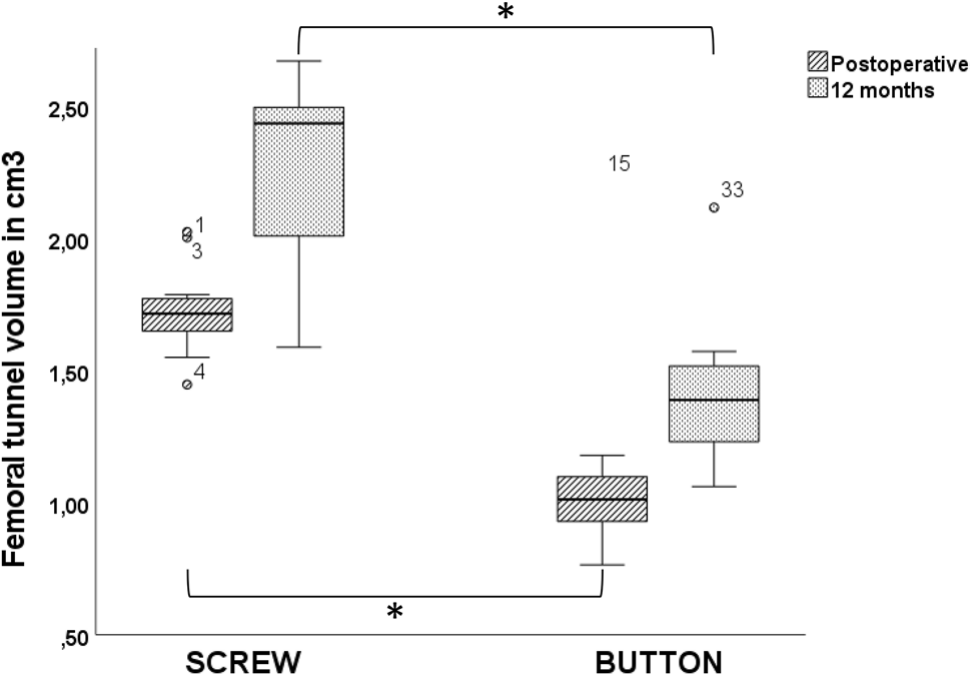
Box plots with medians and interquartile ranges (25–75%) showing femoral tunnel volume in cubic centimeters in the two groups (PEEK, button) in CT scans at different time points (postoperative, 12 months). Significant changes between Screw and Button group were marked with Asterisk (*)

In the group with button fixation, tunnel volume changes showed a mean tunnel enlargement of 111.12 ± 12.72% on the tibial side and 130.96 ± 21.71% on the femoral side. Differences between the two groups were not significant (tibial, *P* = 0.651; femoral, *P* = 0.562) (Table 2, Figs. 1 and 2).

**Table 2:** Results (mean with standard deviation) for anterior cruciate ligament tunnel volume and volume changes

Group	Tibial tunnel (cm^3^)		Femoral tunnel (cm^3^)		Tunnel change (%)	
	Postoperative	12 months	Postoperative	12 months	Tibial	Femoral
Screw	3.12 ± 0.68	3.32 ± 0.57	1.73 ± 0.17	2.14 ± 0.43	108.15 ± 13.7	124.07 ± 25.38
Button	3.05 ± 0.75	3.46 ± 1.34	1.15 ± 0.49	1.44 ± 0.35	111.12 ± 12.72	130.96 ± 21.71
*P*	0.847	0.760	0.002	0.002	0.651	0.562

Patients with additional tibial suture button (N = 10) showed a comparable tunnel volume enlargement of 108.55 ± 12.73% compared with tibial screw fixation without suture button 110.24 ± 14.22% (P = 0.397).

### Tunnel Diameter and Enlargement

**Fig. 3:**
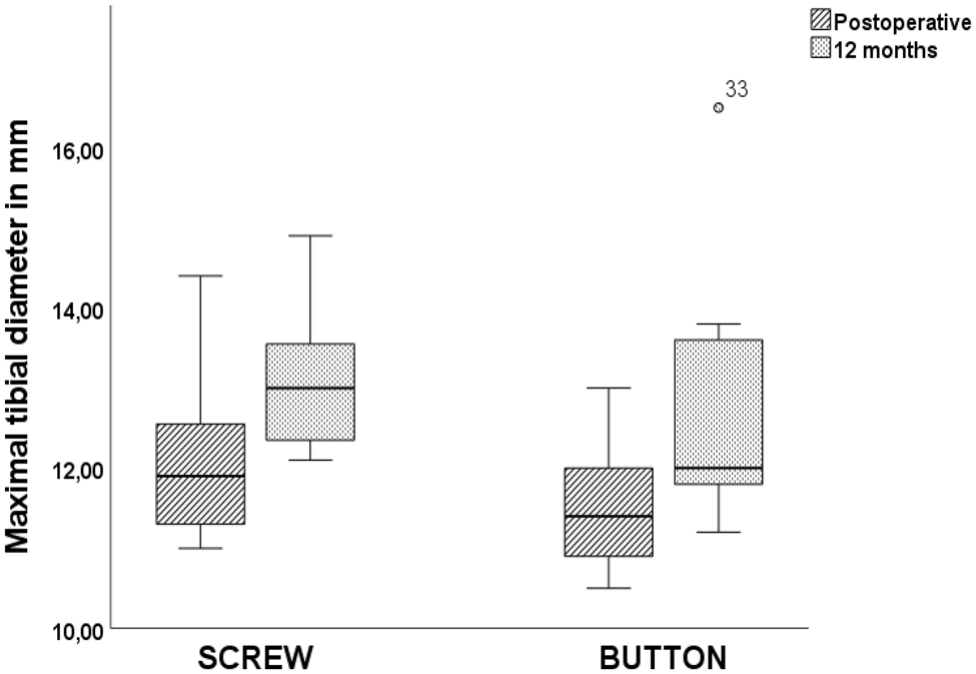
Box plots with medians and interquartile ranges (25–75%) showing maximal tibial tunnel diameters in millimeters in the two groups (PEEK, button) in CT scans at different time points (postoperative, 12 months). It should be noted that PEEK interference screws were used for tibial fixation in both groups

**Fig. 4:**
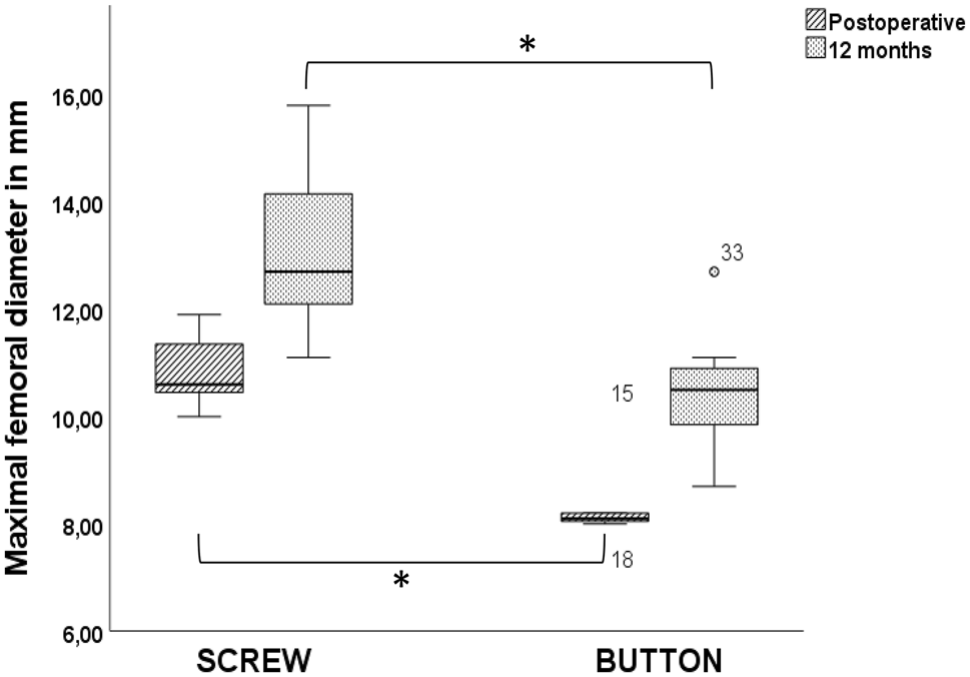
Box plots with medians and interquartile ranges (25–75%) showing maximal femoral tunnel diameter in millimeters in the two groups (PEEK, button) in CT scans at different time points (postoperative, 12 months)**.** Significant changes between Screw and Button group were marked with Asterisk (*)

Femoral tunnels with PEEK screw fixation had significantly larger diameters at time zero and after 12 months in comparison with button fixation (*P* < 0.001, Table 3). Differences in tunnel enlargement, based on the diameter measurements between the two groups, were not significant (tibial, P = 0.317; femoral, *P* = 0.231) (Table 3, Figs. 3 and 4).

**Table 3:** Results (mean with standard deviation) for anterior cruciate ligament maximum tunnel diameter and enlargement

Group	Tibial tunnel (mm)		Femoral tunnel (mm)		Enlargement (%)	
	Postoperative	12 months	Postoperative	12 months	Tibial	Femoral
Screw	12.16 ± 1.11	13.15 ± 0.98	10.88 ± 0.63	13.02 ± 1.43	108.34 ± 5.76	120.05 ± 14.88
Button	11.53 ± 0.87	12.93 ± 1.83	8.19 ± 0.94	10.46 ± 1.29	111.8 ± 8.51	127.92 ± 9.32
*P*	0.219	0.746	< 0.001	< 0.001	0.317	0.231

## Discussion

The present study investigated tibial and femoral tunnel volume changes and maximum diameters after ACL reconstruction using PEEK interference screws and adjustable-length loop cortical button fixation. The most important finding was that PEEK interference screws were associated with significant tibial and femoral tunnel enlargement (rejecting hypothesis 1). Femoral tunnel enlargement was comparable between PEEK interference screw and adjustable-length loop cortical button fixation group (rejecting hypothesis 2).

Interference screws made of PEEK material, an exceptionally strong thermoplastic polymer, are still under investigation for ACL reconstruction. It is been argued that because of their inert nature, PEEK screws are well suited for ACL graft fixation, as they should avoid unpredictable biologic reactions that could lead to tunnel enlargement and osteolysis [15–17, 19, 20]. However, in a recent editorial commentary, Pinczewski and Salmon summarize the disappointing results obtained with biodegradable interference screws, at least in short-term studies [15, 23]. Biodegradable interference screws only showed complete or nearly complete resorption in long-term observations [24]. Although interference screw fixation allows strong graft fixation and early rehabilitation, initial tunnel enlargement is often observed and has been reported in the literature to occur up to 24 months postoperatively [1, 25].

Mayr et al. [1] reported tunnel enlargement with biodegradable interference screws, using volumetric measurements on CT scans after 6 and 24 months postoperatively. The values reported were comparable with the results of the present study, with tunnel volume changes of 112% on the tibial side and 117% on the femoral side. Interestingly, in the present study, PEEK screws now show a tunnel volume increase of 124% on the femoral side and 111% on the tibial side after 12 months, even though the material is considered to be inert. This supports the hypothesis that the enlargement is not due to interaction between the bone and the screw material, but is more probably caused by the tendon-to-bone healing process. In the past, histological and biomechanical investigations have been carried out to obtain data on the phenomenon of tendon–bone healing. Their results suggest that the tendon–bone interface does not mature until graft revascularization and cellular proliferation occurs [26]. This means that graft remodeling precedes soft-tissue healing to bone. It also means that tunnel enlargement does not progress after the remodeling process is over. This might explain why a regression in tunnel volumes has been observed in long-term studies [1]. However, the data presented are of interest for ACL surgeons, since revision ACL reconstructions are often carried out during the first 2 years postoperatively [27].

In the present study, the tibial tunnels were quite large. An important increase in tunnel diameter occurred immediately after surgery with PEEK screw insertion (mean drilled diameter 8.8 ± 0.63 and 8.42 ± 0.58 vs. postoperative diameter 12.16 ± 1.11 and 11.53 ± 0.87). Severely enlarged ACL tunnels have been reported to complicate ACL revision surgery [28]. Two-stage revision surgery needs to be considered when there is tunnel enlargement by more than 10 mm in diameter. Bone refilling is indicated when the bone diameter is larger than 12 mm [28].

Uzumcugil et al. [20] reported a mean tibial diameter change of 118% in anteroposterior radiographs and 119% in lateral radiographs after a mean follow-up period of 28.2 months using PEEK interference screws with soft-tissue grafts. In their study, an alternative screw fixation device in which fixation occurs by inserting a screw in a sheath was adopted. Nevertheless, the results show greater tibial tunnel enlargement in comparison with the present study. The difference could be explained by the use of conventional radiographs for tunnel measurement; conventional radiography has been shown to overestimate the tunnel diameter [21]. In addition, the follow-up time was longer than in the present study. Lind et al. [17] evaluated bone ingrowth with open architecture interference screws in tibial tunnels of patients who had undergone ACL reconstruction with soft-tissue grafts. They found that at 12 months, 42% of the implants showed more than 10% bone ingrowth. They also state that no tunnel widening or cyst formation was observed in any of the patients, although they did not report specific data regarding tunnel diameters. This might be due to the different interference screw design used in that study, while in the present study nonporous screws were used. However, comparison between tunnel enlargements with different PEEK interference screw designs might be of interest. Another research group compared clinical and radiological outcomes with PEEK and titanium interference screws for ACL reconstruction [19]. They reported that equivalent tunnel volumes were measured on MRI 1 year after surgery. However, as the clinical outcome was the primary object, tunnel diameters were only measured at the follow-up examination, without baseline measurements; it was therefore not possible to detect tunnel enlargement. To date, the present study is the first to report on tunnel enlargement after ACL reconstruction with PEEK interference screws using CT volumetric measurements.

The study is also the first to compare adjustable-length loop cortical button fixation with PEEK interference screws in relation to tunnel enlargement. No significant differences in femoral tunnel volume changes 12 months after operation were found between the two fixation techniques in the present study. However, tunnel diameters were significantly larger postoperatively with screw fixation in comparison with button fixation (10.88 ± 0.63 mm vs. 8.19 ± 0.94 mm). In general, interference screw insertion results in initial tunnel expansion, and surgeons tend to drill smaller tunnels with button fixation with the press-fit technique. Finally, femoral tunnels with PEEK screw fixation were significantly larger in comparison with button fixation at 12 months postoperatively (13.02 ± 1.43 mm vs. 10.46 ± 1.29 mm). The risk of a need for staged revision surgery might, therefore, be greater after femoral PEEK screw fixation in comparison with button fixation.

The present study has some limitations. First, only a total of 18 patients were included and no power analysis was conducted previously. These limitations are owed to the retrospective study design of our study. In addition, the patients were not assigned to the study groups randomly. However, differences in femoral tunnel enlargement were highly significant between the two groups. It should also be mentioned that this study only investigated tunnel diameter and volume changes, without reporting on the clinical outcomes for the patients included. Furthermore, in ten patients, an additional tibial extracortical fixation was performed. However, tunnel enlargement was not different for tibial interference screw with or without use of additional suture button. Changes in the tunnel volume that occur later than 1 year postoperatively were not investigated by the present study. However, ACL revisions are frequently observed within the first year after primary ACL reconstruction [27].

One strength of the study is the use of CT scanning for volumetric measurement of tunnel enlargement. High-resolution CT scanning was used to evaluate the tibial and femoral tunnel volume and diameter postoperatively and at 12 months after surgery. CT scanning has been reported to be the gold standard and the most accurate image modality for assessing tunnel volumes and diameters following ACL reconstruction [1, 2, 21, 22]. It should also be noted that the behavior of PEEK interference screws after ACL reconstruction has not often been investigated yet [17, 19], as the majority of materials research on PEEK has been in nonorthopedic fields.

To the best of the authors’ knowledge, this is the first trial that has reported tunnel enlargement and volume changes after ACL reconstruction with PEEK interference screws in comparison with adjustable cortical button fixation using CT scan measurements.

## Conclusion

The present study has shown that the use of PEEK interference screws is associated with significant tibial and femoral tunnel enlargement. Femoral tunnel enlargement was comparable between PEEK interference screws and adjustable-length loop cortical button fixation. However, the final tunnels after 12 months were significantly larger with PEEK screw fixation than with adjustable-length loop cortical button fixation.

## Data Availability

The manuscript was not previously submitted for publication to any other Journal.

## References

[1] Mayr R (2020). ACL reconstruction with adjustable-length loop cortical button fixation results in less tibial tunnel widening compared with interference screw fixation. Knee Surgery, Sports Traumatology, Arthroscopy.

[2] Mayr R (2017). Tunnel widening after ACL reconstruction with aperture screw fixation or all-inside reconstruction with suspensory cortical button fixation: Volumetric measurements on CT and MRI scans. The Knee.

[3] Fink C (2001). Tibial tunnel enlargement following anterior cruciate ligament reconstruction with patellar tendon autograft. Arthroscopy.

[4] Clatworthy MG (1999). Tunnel widening in anterior cruciate ligament reconstruction: A prospective evaluation of hamstring and patella tendon grafts. Knee Surgery, Sports Traumatology, Arthroscopy.

[5] Kamath GV (2011). Revision anterior cruciate ligament reconstruction. American Journal of Sports Medicine.

[6] Rodeo SA (2018). Editorial commentary: the quest to prevent knee anterior cruciate ligament bone tunnel widening continues. Arthroscopy.

[7] Taketomi S (2021). Editorial commentary: tunnel widening after anterior cruciate ligament reconstruction may increase laxity and complicate revision. Arthroscopy.

[8] Lubowitz JH, Schwartzberg R, Smith P (2013). Randomized controlled trial comparing all-inside anterior cruciate ligament reconstruction technique with anterior cruciate ligament reconstruction with a full tibial tunnel. Arthroscopy.

[9] Barrow AE (2014). Femoral suspension devices for anterior cruciate ligament reconstruction: Do adjustable loops lengthen?. American Journal of Sports Medicine.

[10] Mayr R (2015). Biomechanical comparison of 2 anterior cruciate ligament graft preparation techniques for tibial fixation: Adjustable-length loop cortical button or interference screw. American Journal of Sports Medicine.

[11] Houck DA (2018). Fixed- versus adjustable-loop femoral cortical suspension devices for anterior cruciate ligament reconstruction: a systematic review and meta-analysis of biomechanical studies. Orthopaedic Journal of Sports Medicine.

[12] Lubowitz JH, Schwartzberg R, Smith P (2015). Cortical suspensory button versus aperture interference screw fixation for knee anterior cruciate ligament soft-tissue allograft: a prospective randomized controlled trial. Arthroscopy.

[13] Kaeding C (2005). A prospective randomized comparison of bioabsorbable and titanium anterior cruciate ligament interference screws. Arthroscopy.

[14] Bourke HE (2013). Randomized controlled trial of osteoconductive fixation screws for anterior cruciate ligament reconstruction: A comparison of the Calaxo and Milagro screws. Arthroscopy.

[15] Pinczewski LA, Salmon LJ (2017). Editorial commentary: the acrid bioscrew in anterior cruciate ligament reconstruction of the knee. Arthroscopy.

[16] Kurtz SM, Devine JN (2007). PEEK biomaterials in trauma, orthopedic, and spinal implants. Biomaterials.

[17] Lind M (2020). Bone ingrowth into open architecture PEEK interference screw after ACL reconstruction. J Exp Orthop.

[18] Fu L (2021). Grafting polymer brushes by ATRP from functionalized poly (ether ether ketone) microparticles. Polymers for Advanced Technologies.

[19] Shumborski S (2019). A Randomized controlled trial of PEEK versus titanium interference screws for anterior cruciate ligament reconstruction with 2-year follow-up. American Journal of Sports Medicine.

[20] Uzumcugil O (2012). Effect of PEEK polymer on tunnel widening after hamstring ACL reconstruction. Orthopedics.

[21] Marchant MH (2010). Comparison of plain radiography, computed tomography, and magnetic resonance imaging in the evaluation of bone tunnel widening after anterior cruciate ligament reconstruction. Knee Surgery, Sports Traumatology, Arthroscopy.

[22] Foldager C (2010). Tibial tunnel widening after bioresorbable poly-lactide calcium carbonate interference screw usage in ACL reconstruction. Knee Surgery, Sports Traumatology, Arthroscopy.

[23] Kiekara T (2017). Femoral and tibial tunnel diameter and bioabsorbable screw findings after double-bundle ACL reconstruction in 5-year clinical and MRI follow-up. Orthopaedic journal of sports medicine.

[24] Sundaraj K (2020). Bioabsorbable Versus Titanium Screws in Anterior Cruciate Ligament Reconstruction Using Hamstring Autograft: A Prospective, Randomized Controlled Trial With 13-Year Follow-up. American Journal of Sports Medicine.

[25] Putnis SE (2021). Adjustable suspension versus hybrid fixation in hamstring autograft anterior cruciate ligament reconstruction. The Knee.

[26] Rodeo SA (1993). Tendon-healing in a bone tunnel. A biomechanical and histological study in the dog. Journal of Bone and Joint Surgery. American Volume.

[27] Schmücker M (2021). Graft failure, revision ACLR, and reoperation rates after ACLR with quadriceps tendon versus hamstring tendon autografts: a registry study with review of 475 patients. American Journal of Sports Medicine.

[28] Mayr R (2012). Revision anterior cruciate ligament reconstruction: an update. Archives of Orthopaedic and Trauma Surgery.

